# Application value of resting-state fMRI in preoperative lateralization of language areas in epilepsy with left-sided epileptogenic foci

**DOI:** 10.3389/fneur.2026.1839830

**Published:** 2026-06-10

**Authors:** Zihao Wang, Tianqi Hu, Meng Wang, Ying Li, Yaning Sun, Di Zhang, Wenling Li

**Affiliations:** 1 Department of Neurosurgery, The Second Hospital of Hebei Medical University, Shijiazhuang, China; 2 Department of Neurosurgery, Beijing Tiantan Hospital, Capital Medical University, Beijing, China; 3 Children’s Hospital of Hebei Province, Shijiazhuang, China

**Keywords:** epilepsy, language area, lateralization, left-sided epileptogenic focus, resting-state functional magnetic resonance imaging, visualized evaluation

## Abstract

**Objective:**

Focusing on patients with epilepsy and left-sided epileptogenic foci, this study aimed to clarify language lateralization differences from healthy individuals, explore Broca’s/Wernicke’s area lateralization and dominant hemisphere shift, and construct a laterality index–activation map combined scheme. It provides a theoretical basis for optimizing epilepsy surgery and reducing postoperative language impairment risk.

**Methods:**

We retrospectively studied 36 patients with left-sided epileptogenic foci and 45 healthy controls (2018–2023, Hebei Medical University Second Hospital). After preprocessing rs-fMRI data, 12 bilateral language-related seed points were selected to calculate functional connectivity and generate activation maps. We further computed two types of laterality indexes: the global laterality index (LI) reflecting overall whole-brain language lateralization, and the regional LI assessing independent lateralization of Broca’s area (6 frontal seed points) and Wernicke’s area (6 temporal seed points). The Kappa coefficient was used to analyze the consistency between different methods, and SEEG cortical stimulation (only performed in surgical candidates) combined with postoperative follow-up was applied to verify clinical reliability.

**Results:**

Non-classical language dominance was higher in epilepsy (69.4% vs. 45.5%, *p* = 0.024), especially in Wernicke’s area (81.8% vs. Broca’s 50.0%). Laterality index–activation map consistency was 83.3% (Kappa = 0.586); regional laterality index–activation map consistency was 75.0% (Kappa = 0.40). SEEG stimulation and surgical verification confirmed that the left Broca’s area retained core language function, whereas the left Wernicke’s area showed significant functional impairment (only 10.0% positive stimulation rate, and no language deficits occurred after partial resection in 6 patients).

**Conclusion:**

Left-sided epilepsy patients show language lateralization remodeling, with Wernicke’s area more prone to shift. Bilateral language dominance does not equate to equal functional contribution; the left Broca’s area retains core motor language function and requires priority protection in all epilepsy surgeries involving the left frontotemporal lobe, especially left frontal lobe epileptogenic focus resection. The combined laterality index–activation map scheme reliably supports preoperative language lateralization, optimizes surgery, and reduces language impairment risk, with clinical value.

## 1 Introduction

Language function depends on the synergistic effect of key brain regions such as Broca’s area and Wernicke’s area (1). In epilepsy patients, especially those with lesions involving the frontotemporal lobe of the dominant hemisphere (2, 3), the pattern of language lateralization often changes significantly. Temporal lobe epilepsy (TLE) and frontal lobe epilepsy (FLE) together account for 60–70% of all epilepsy cases. For such patients, the risk of language function impairment during surgical resection of epileptogenic foci is significantly increased due to the close anatomical proximity between epileptogenic foci and Broca’s/Wernicke’s areas (4–6). For patients with drug-resistant epilepsy, surgical resection of epileptogenic foci is the main means to achieve clinical cure at present (7). However, due to the lack of reliable anatomical markers for localizing key language regions in human brain structural images (8), irreversible language function deficits are likely to occur after surgery if the functional lateralization characteristics of language-related brain regions are not accurately evaluated preoperatively.

At present, the gold standard for clinical detection of language dominant hemisphere is the Wada test, but this method is an invasive examination with the risk of inducing complications and a complex operation process, which limits its clinical application. Functional magnetic resonance imaging (fMRI), as a non-invasive brain function assessment method, has been gradually applied to the preoperative evaluation of language lateralization in epilepsy (9). Task-based fMRI is the first to be widely used, and its consistency with the results of the Wada test exceeds 80% (10), which is supported by the guidelines of the Organization for Human Brain Mapping (OHBM) and the American Academy of Neurology (AAN) (11). Benjamin et al. found that almost all epilepsy centers used fMRI for language lateralization analysis, and most use a 10 mm distance to guide the surgical boundary (12). However, task-based detection is time-consuming and has high compliance requirements, making it difficult to carry out on a large scale. The popularization of resting-state fMRI (rs-fMRI) has addressed this issue. Rs-fMRI has been proven to be applicable to the preoperative evaluation of language lateralization in epilepsy patients (13), and the American Society of Neuroradiology has recommended standardized rs-fMRI acquisition and preprocessing steps (14), which significantly improve the reliability and repeatability of the results. A survey showed that 96% of patients prefer to determine the language dominant hemisphere by rs-fMRI compared with the invasive Wada test and compliance-dependent task-based fMRI (12), and many scholars believe that rs-fMRI has emerged as a promising alternative to task-based language lateralization detection (15–19). A team study confirmed that the accuracy of rs-fMRI consistent with the Wada test results is over 80% (20, 21).

With the in-depth research, the refinement of language area evaluation has become a new development trend. Previous studies have shown that partition analysis of Broca’s area and Wernicke’s area can more accurately reflect the changes in language function of epilepsy patients: Jansen, Andreas et al. (22) proposed crossed dominance of language areas using task-based fMRI. Lorenzo Caciagli et al. focused on the influence of epilepsy types and found that FLE mainly affects the function of Broca’s area and the default mode network (DMN), while TLE mainly affects the function of Wernicke’s area (1). In addition, Karami, M et al. divided 15 classification methods for language areas (23), and other teams also used similar methods (24).

Existing studies still have some aspects that can be further improved: (1) Most of the participants include patients with bilateral epileptogenic foci, without focusing on the specific impact of left-sided epileptogenic foci; (2) Many studies prioritize quantitative evaluation using the laterality index (LI) and pay insufficient attention to the clinical utility of visualized activation maps, resulting in the generated maps failing to provide actionable guidance for surgical planning (9, 15). Conversely, studies that focus primarily on visualized activation maps for qualitative assessment often lack systematic and detailed quantitative analysis of lateralization coefficients (17). These two distinct methodological biases may independently or jointly affect the accuracy of preoperative language lateralization assessment and introduce uncertainties into surgical decision-making. (3) A complete joint verification system has not been established, so the accuracy and clinical applicability of the evaluation still have room for improvement.

Based on the above background, this study focused on patients with left-sided epileptogenic foci and defined three core research priorities. First, we aimed to clarify the differences in language lateralization between these patients and healthy individuals, and explore the regional lateralization characteristics of Broca’s and Wernicke’s areas as well as the inter-regional differences in dominant hemisphere shift tendency. Second, we sought to construct a combined evaluation scheme integrating quantitative laterality indexes and qualitative visualized activation maps. The primary outcomes of this study included the characterization of region-specific language remodeling patterns in left-sided epilepsy and the verification of the consistency and clinical reliability of the combined evaluation scheme. Ultimately, this study aimed to provide a solid theoretical basis and actionable clinical reference for optimizing individualized epilepsy surgical procedures and reducing the risk of permanent postoperative language impairment.

## 2 Methods

### 2.1 Study subject selection

This retrospective study was approved by the Ethics Review Committee of the Second Hospital of Hebei Medical University (Approval No.: 2023-R643), and written informed consent was obtained from all subjects and their family members. Epilepsy patients with left-sided epileptogenic foci and healthy subjects who underwent rs-fMRI examination in our hospital from January 2018 to December 2023 were enrolled and divided into the epilepsy group and the healthy group.

Inclusion criteria for the epilepsy group: (1) Diagnosed as focal epilepsy according to the 2017 International League Against Epilepsy (ILAE) epilepsy classification criteria; (2) Determined to have single or multiple left-sided epileptogenic foci by comprehensive evaluation including long-term video electroencephalogram, structural MRI, PET and SEEG; (3) With the willingness of surgical treatment, complete preoperative evaluation and complete clinical data; (4) Maintained stable epileptic conditions with well-controlled and relatively consistent seizure frequency at the time of scanning; (5) Able to cooperate with rs-fMRI scanning and cognitive function evaluation.

Exclusion criteria for the epilepsy group: (1) Patients with generalized epilepsy or unclear localization of epileptogenic foci; (2) Patients complicated with other nervous system diseases such as traumatic brain injury, cerebrovascular disease and brain tumor; (3) Patients with contraindications to MRI scanning; (4) Patients with severe cognitive dysfunction who cannot cooperate with the examination; (5) Patients with a bilingual background.

Inclusion criteria for the healthy group: (1) No history of epilepsy and other nervous system diseases, and no family history of related diseases; (2) Right-handed with normal cognitive function; (3) Able to cooperate with rs-fMRI scanning.

Exclusion criteria for the healthy group: (1) Subjects with brain structural or functional abnormalities; (2) Subjects with a history of mental illness or substance abuse; (3) Bilingual individuals were also excluded.

Demographic characteristics (age, gender, handedness) of all subjects were collected, in which handedness was evaluated by the Edinburgh Handedness Inventory; clinical data of patients in the epilepsy group were also collected, including disease course, average monthly seizure frequency, location of epileptogenic foci and surgical method.

### 2.2 Acquisition and preprocessing of MRI data

All subjects underwent MRI scanning using a Philips Achieva 3.0 T (16-channel coil) or General Electric (GE) SIGNA Architect 3.0 T (48-channel coil) magnetic resonance scanner. Before scanning, subjects were instructed to keep their eyes closed and awake with their heads fixed to reduce motion artifacts; if an epileptic seizure occurred during scanning, the scanning was stopped immediately and the data were re-collected after the patient’s condition stabilized.

Scanning parameters: (1) Philips device: T1-weighted structural image (sagittal): TR = 7.6 ms, TE = 3.7 ms, FA = 8°, slice number = 180, slice thickness = 1.0 mm, FOV = 230 mm × 180 mm, voxel size = 1.0 mm × 1.0 mm × 2.0 mm; rs-fMRI sequence (GRE-EPI): TR = 2000 ms, TE = 30 ms, FA = 90°, FOV = 220 mm × 220 mm, matrix = 72 × 73, slice number = 32, slice thickness = 4 mm (no interslice gap), voxel size = 3.0 mm × 3.0 mm × 4.0 mm, scanning direction parallel to the gyrus rectus. (2) GE device: T1-weighted structural image (sagittal): TR = 6.8 ms, TE = 2.7 ms, TI = 600 ms, FA = 8°, slice thickness = 0.5 mm, FOV = 220 mm × 200 mm, voxel size = 1.0 mm × 1.0 mm × 1.0 mm; rs-fMRI sequence (GRE-EPI): TR = 3,000 ms, TE = 35 ms, FA = 90°, FOV = 220 mm × 220 mm, matrix = 80 × 80, slice number = 36, slice thickness = 4 mm (no interslice gap), voxel size = 2.8 mm × 2.8 mm × 4.0 mm, scanning direction parallel to the gyrus rectus.

To minimize batch effects from scanner differences, standard procedures were implemented: core rs-fMRI parameters were matched as closely as possible, all data were resampled to 2 mm cubic voxels to unify spatial resolution, and the standard ComBat method was applied for data harmonization. Scanner type was included as a covariate in all statistical analyses to control for residual differences, and no systematic device-related bias affecting study conclusions was identified.

Data preprocessing was completed on the MATLAB R2017a platform using self-developed code (25)(available from the corresponding author), combined with SPM12 (r7771) and FSL 6.0 software. The specific steps were as follows: (1) Discarding the first 4 volume data to eliminate the T1 equilibrium effect; (2) Correcting the interslice acquisition time offset using SPM12; (3) Completing rigid body translation/rotation through FSL to correct head motion artifacts, and excluding subjects with head displacement >1.5 mm or rotation >1.5°; (4) Resampling to 2 mm cubic voxels to unify the spatial resolution; (5) Time filtering to retain low-frequency signals below 0.08 Hz and remove constant offset and linear trend; (6) Performing spatial smoothing with a 6 mm full width at half maximum (FWHM) Gaussian kernel; (7) Confounding variable regression to remove the interference of 6 motion parameters obtained from head motion correction, whole-brain average signal, lateral ventricular signal and deep white matter signal.

### 2.3 Functional connectivity and visualization method of language-related seed points

**Figure 1:**
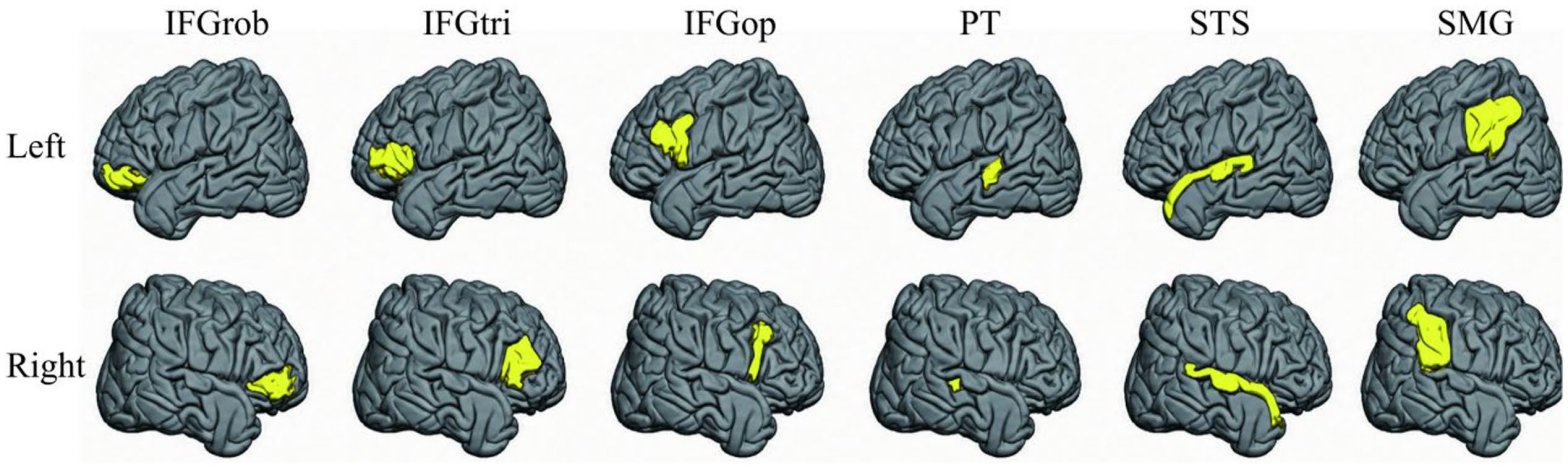
All 12 seed points were defined as 6 mm radius spherical ROIs based on the Desikan-Killiany cortical atlas. The exact MNI coordinates are as follows: left hemisphere [−42, 32, −12], [−48, 20, 5], [−45, 10, 25], [−52, −32, 10], [−55, −40, 5], [−50, −45, 30]; right hemisphere [42, 32, −12], [48, 20, 5], [45, 10, 25], [52, −32, 10], [55, −40, 5], [50, −45, 30]. Left: Left cerebral hemisphere; Right: Right cerebral hemisphere; IFGorb: Inferior frontal gyrus, orbital part; IFGtri: Inferior frontal gyrus, triangular part; IFGop: Inferior frontal gyrus, opercular part; PT: Planum temporale; STS: Superior temporal sulcus; SMG: Supramarginal gyrus.

Based on the anatomical localization of the language functional network (26–28), 12 language-related seed points were selected in the bilateral frontotemporal lobes, covering the key subregions of language function and being bilaterally symmetric, including: orbital part, triangular part, opercular part, planum temporale, superior temporal sulcus, supramarginal gyrus (6 points on each side). All seed points were defined as 6 mm radius spherical ROIs based on the Desikan-Killiany cortical atlas; their exact MNI coordinates are provided in the legend of Figure 1.

A weighted linear summation integration strategy was adopted for data processing to avoid the one-sidedness of a single seed point. First, the average time series of all voxels in each seed point region was extracted, and the Pearson correlation coefficient between this series and the time series of all other vertices on the cerebral cortex surface was calculated to generate a whole-brain functional connectivity map (25). Weight assignment was based on prior knowledge to highlight the contribution of core regions that are more critical to language function. In the integration process, only positive functional connectivity values were retained, and all negative correlation connections were set to zero to focus on brain regions with synergistic activities. The integrated functional connectivity map was nonlinearly registered back to the subject’s native anatomical space from the standard space (fsaverage4) to adapt to the individualized localization needs of clinical surgical planning; finally, the tksurfer tool in the FreeSurfer software package was used to superimpose the connectivity strength in pseudocolor and visualize it on the 3D cortical surface reconstruction model. The schematic diagram of the 12 seed regions is shown in Figure 1.

### 2.4 Visualized activation maps of language areas

**Figure 2:**
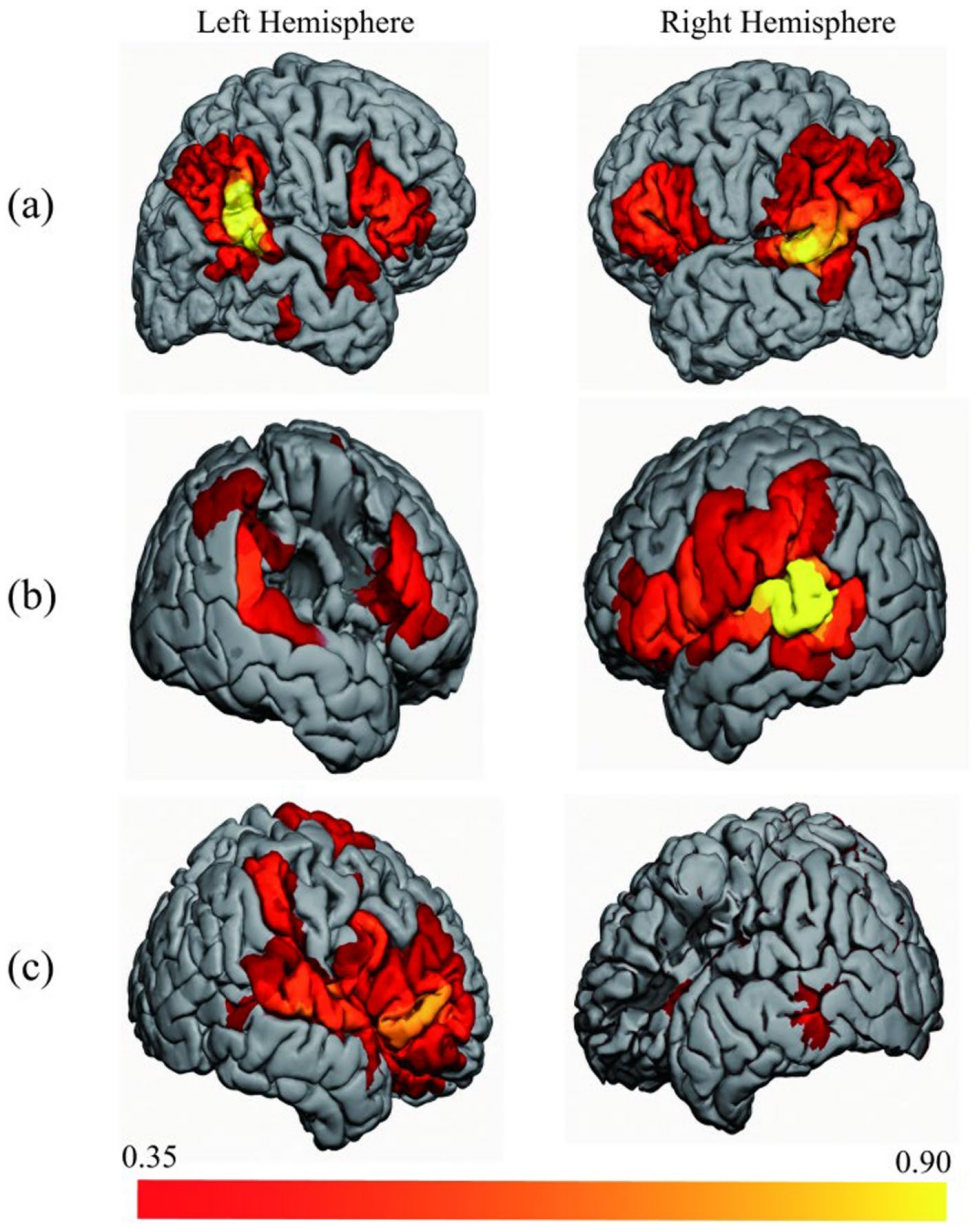
Resting-state functional connectivity maps of the language network under different language dominance patterns: **(a)** Bilateral linguistic dominance, **(b)** Right hemispheric linguistic dominance, **(c)** Left hemispheric linguistic dominance. The standardized heatmap color bar ranges from 0.35 to 0.9, representing statistically significant positive functional connectivity strength between brain regions and predefined language seed points, with brighter (yellow) colors indicating stronger connectivity. Only voxels with connectivity values above the threshold of 0.35 are displayed.

Visualized activation maps of language areas were established. Two attending clinicians with more than 10 years of experience in neuroimaging and preoperative evaluation of epilepsy surgery independently evaluated the visualized activation maps in a blinded manner. Unified training and standardized judgment criteria were formulated before the evaluation: 1. If the highlight degree of the target area in one cerebral hemisphere is significantly higher than that in the homotopic area of the contralateral side, the side is determined to be the language dominant hemisphere; 2. If the highlight degree and range of the bilateral sides are basically the same, it is determined to be bilateral language dominance. The judgment results of the visualized activation maps were regarded as the “gold standard” shown in Figure 2.

### 2.5 Laterality index and regional laterality index

Formula for calculating the laterality index (LI): LI = (LSLH-RSRH)/(LSLH+RSRH), where LSLH is the number of voxels showing significant functional connectivity between the 6 left seed points and the left hemisphere, and RSRH is the voxel count of functional connectivity between 6 right seed points and the right hemisphere. The value range of LI is −1 to 1, and the judgment criteria are as follows: LI ≥ 0.20 for left language dominance, LI ≤ -0.20 for right language dominance, and −0.20 < LI < 0.20 for bilateral language dominance. The threshold selection was determined by multi-threshold iterative verification: the LI results under the thresholds of 0.5, 0.7, 0.9, 0.95 and 0.98 were calculated in the pre-experiment. It was found that the voxel count was too high when the threshold was <0.7, and the LI tended to be bilateral; effective data could not be obtained for some subjects due to weak functional connectivity when the threshold was >0.95; the LI value had the best stability and the highest consistency with the visualized activation maps when the threshold was 0.9, so the calculation results with the threshold of 0.9 were finally selected.

**Figure 3:**
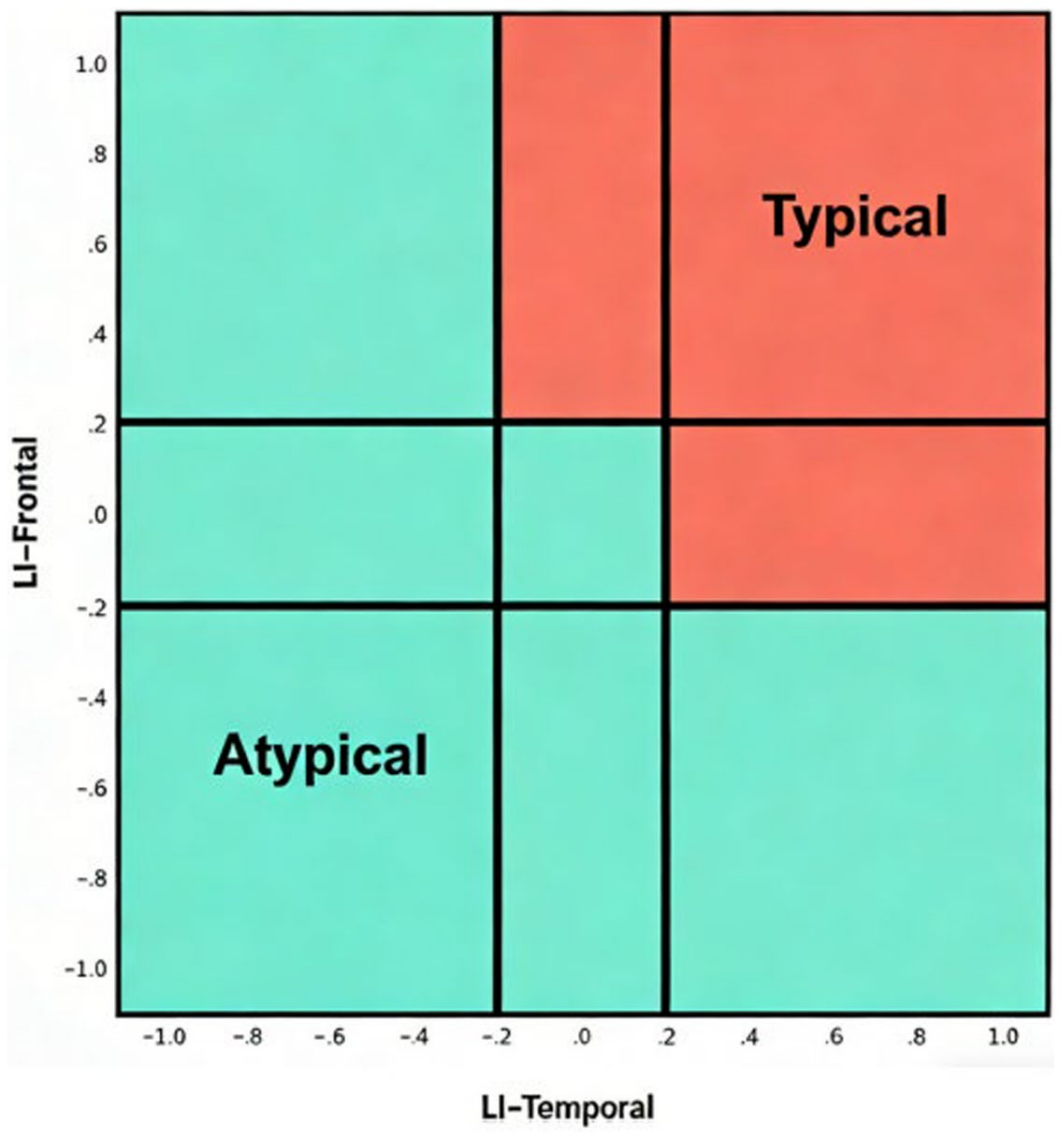
Language pattern definition. The overall language pattern is defined based on the laterality indexes for frontal and temporal language regions. The typical pattern is depicted in red, and the atypical pattern is in cyan.

The core language areas were divided into Broca’s area (frontal lobe region of interest, ROI) and Wernicke’s area (temporal lobe ROI), with 6 seed points in each of the bilateral frontal and temporal lobe ROIs. The voxel count of functional connectivity between the seed points of each partition and the corresponding hemisphere was calculated respectively, and the regional LI was calculated according to the above formula with the same judgment criteria as LI. Language dominant hemisphere was divided into classical dominance and non-dominant (Figure 3) (23). The results obtained from LI and regional laterality index were used to evaluate the consistency with the visualized analysis results.

### 2.6 SEEG electrical stimulation and Resective surgery verification

To verify the reliability of fMRI visualized activation maps, SEEG cortical lateral electrode sites covering the highlighted areas of fMRI language dominant hemisphere were selected during the localization of epileptogenic foci. The electrical stimulation parameters were set as follows: current intensity 1–5 mA, stimulation frequency 50 Hz, single stimulation duration 5 s, and stimulation interval not less than 10 s. Electrical stimulation was performed while the patients completed language tasks such as listening, speaking, reading and writing periodically, and involuntary interruption of language tasks was taken as the positive judgment criterion for electrode sites. For patients with epileptogenic foci adjacent to the traditional Broca’s area and Wernicke’s area, surgical plans were formulated preoperatively according to the evaluation results of fMRI language dominant hemisphere, and partial traditional language areas were resected during surgery. Postoperative language function was assessed using the Western Aphasia Battery (WAB-R) at 1 week, 1 month, and 3 months after surgery to verify the accuracy of the preoperative fMRI visualization results.

### 2.7 Statistical analysis

Continuous variables conforming to normal distribution were expressed as mean ± standard deviation (SD), skewed continuous variables as median (interquartile range), and categorical variables as counts and percentages. Pearson’s chi-square test was used to compare the differences in the distribution of language dominant hemisphere between independent groups; paired chi-square test (McNemar test) was used to analyze the differences in the classification results of language dominant hemisphere between the two rs-fMRI methods (laterality index, regional laterality index) and visualized activation maps in the same cohort. Kappa consistency coefficient was used to quantify the consistency strength of laterality index, regional laterality index and visualized activation maps in the classification of language dominant hemisphere. SPSS 27 and R 4.5.2 software were used for statistical analysis.

## 3 Results

### 3.1 Characteristics of study subjects

A total of 36 epilepsy patients and 45 healthy subjects were enrolled in this study. The demographic characteristics of the two groups, clinical disease characteristics of epilepsy patients and language dominant hemisphere results detected by different methods are as follows. In terms of demographic characteristics, the median age of epilepsy patients was 20.5 years, 27 cases were male (75.0%), and 29 cases were right-handed (80.6%); the median age of healthy subjects was 25.0 years, 19 cases were male (42.2%), and all were right-handed (100%). The median disease course of epilepsy patients was 6.0 years, and the median average monthly seizure frequency was 0.3 times; Epileptogenic foci were distributed into two main anatomical categories: the temporal, parietal, occipital, and insular lobes (22 cases, 61.1%) and the frontal lobe (14 cases, 38.9%); there were differences in surgical treatment methods: 15 patients underwent SEEG combined with resective surgery (41.7%), 14 underwent resective surgery (38.9%), 4 underwent only SEEG (11.1%), and another 3 did not receive surgical treatment (8.3%).

The proportion of left hemisphere language dominance detected by different methods was as follows: 11 cases (30.6%) in the epilepsy group and 25 cases (55.5%) in the healthy group by the visualized activation map method; 9 cases (25.0%) in the epilepsy group and 17 cases (37.8%) in the healthy group by the overall laterality index method; by the regional laterality index method, 14 cases (39.9%) in the epilepsy group had left dominance in Broca’s area and 7 cases (19.4%) had left dominance in Wernicke’s area, and no partition detection was performed in the healthy group. The basic characteristics of the study subjects are shown in Table 1.

**Table 1:** Basic information of the study subjects.

	Epilepsy patients (*n* = 36)	Healthy subjects (*n* = 45)
Age (y)	20.5 (14.0,27.0)	25.0 (24.0,31.5)
Gender (male, %)	27 (75.0%)	19 (42.2%)
Handedness (right-handed, %)	29 (80.6%)	45 (100%)
Epilepsy course (y)	6.0 (3.0,15.0)	–
Average monthly seizure frequency	0.3 (0.2,3.5)	–
Seizure onset area, *n* (%)		
Frontal lobe	14 (38.9%)	–
Temporal/ parietal/ occipital/ insular lobe	22 (61.1%)	–
Surgical method, *n* (%)		
SEEG + resective surgery	15 (41.7%)	–
SEEG only	4 (11.1%)	–
Resective surgery only	14 (38.9%)	–
No surgery	3 (8.3%)	–
Language dominant hemisphere (left, %) by visualized activation map	11 (30.6%)	25 (55.5%)
Language dominant hemisphere (left, %) by laterality index	9 (25.0%)	17 (37.8%)
Language dominant hemisphere (left, %) in Broca’s area by regional laterality index	14 (39.9%)	–
Language dominant hemisphere (left, %) in Wernicke’s area by regional laterality index	7 (19.4%)	–

### 3.2 Analysis of language dominance characteristics and handedness correlation in epilepsy patients with left-sided epileptogenic foci and healthy subjects

The results of visualized activation maps showed that 11 cases (30.6%) in the epilepsy group had classical language dominance, and 25 cases (69.4%) had non-classical language dominance. Among healthy subjects, 25 cases (55.5%) had classical language dominance and 20 cases (45.5%) had non-classical language dominance. Pearson’s chi-square test showed that the difference in the distribution of language dominant hemisphere between the two groups was statistically significant (*p* = 0.024), suggesting that epilepsy patients with left-sided epileptogenic foci are more likely to have non-classical language dominance.

**Figure 4:**
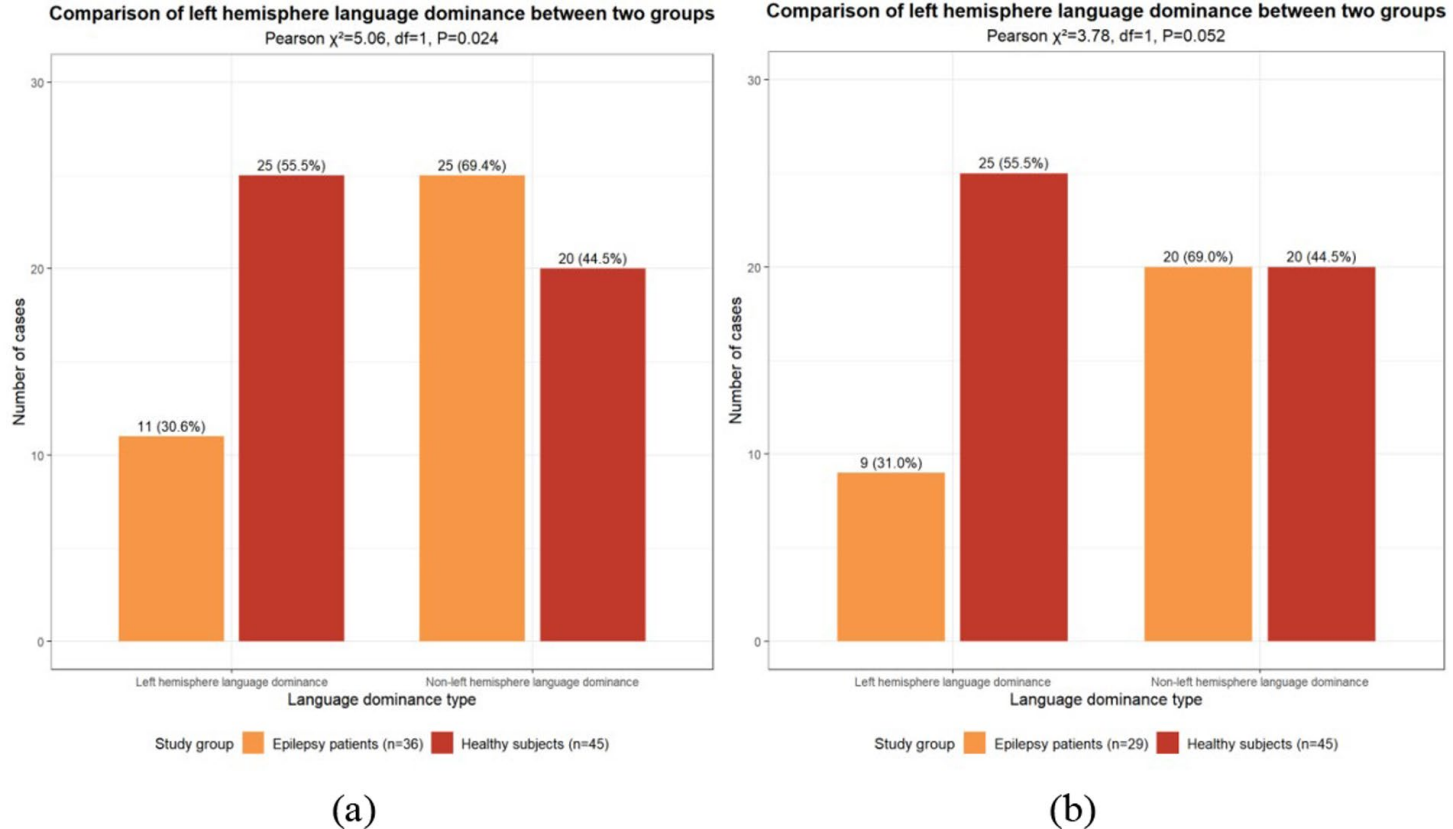
Language dominance characteristics in epilepsy patients and healthy subjects.

Handedness correlation analysis showed that among 29 right-handed patients in the epilepsy group, 9 cases (31.0%) had left classical language dominance and 20 cases (69.0%) had non-dominant; among 45 right-handed subjects in the healthy group, 25 cases (55.5%) had left classical language dominance and 20 cases (44.5%) had non-dominant. Pearson’s chi-square test showed that there was no statistically significant difference in the distribution of language dominant hemisphere between the right-handed populations of the two groups (*χ*^2^ = 3.79, df = 1, *p* = 0.052), but there was a potential trend of a higher proportion of non-dominant in the epilepsy group (Figure 4).

### 3.3 Consistency evaluation of language dominance between laterality index/regional laterality index and visualized activation maps

The judgment of language dominant hemisphere in patients based on visualized activation maps (gold standard) showed that 11 cases (30.6%) had classical left language dominance and 25 cases (69.4%) had non-dominant (including right/bilateral dominance); the judgment results based on the laterality index showed that 9 cases (25.0%) suggested left language dominance and 27 cases (75.0%) had non-dominant. McNemar test showed that there was no statistically significant difference between the judgment results of the laterality index and visualized activation maps (*p* = 0.317). Consistency test showed that the Cohen’s Kappa value of LI and visualized activation maps was 0.586, indicating a moderate consistency; the overall consistency was 83.3%, the sensitivity (positive identification rate) was 92.0%, and the specificity (negative identification rate) was 63.6%.

The consistency between the regional laterality index and visualized activation maps was analyzed by grouping according to the location of epileptogenic foci. For left frontal lobe epileptogenic foci (*n* = 14): visualized activation maps showed 7 cases (50.0%) with left dominance in Broca’s area and 7 cases (50.0%) with non-dominant; the judgment results of the regional laterality index showed 7 cases (50.0%) with left dominance in Broca’s area and 7 cases (50.0%) with non-dominant. For left temporal–parietal-occipital-insular lobe epileptogenic foci (*n* = 22): visualized activation maps showed 4 cases (18.2%) with left dominance in Wernicke’s area and 18 cases (81.8%) with non-dominant; the judgment results of the regional laterality index showed 3 cases (13.6%) with left dominance in Wernicke’s area and 19 cases (86.4%) with non-dominant. The Kappa consistency coefficient reached 0.40, indicating a moderate consistency; the overall consistency was 75.0%, the sensitivity (positive identification rate) was 84.0%, and the specificity (negative identification rate) was 54.5% (Table 2).

**Table 2:** Consistency comparison of language lateralization evaluation methods.

	Evaluation method pair	Consistency rate sensitivity	Specificity	Kappa consistency coefficient
Laterality index vs. Visualized activation map	83.3%	92.0%	63.6%	0.59
Regional laterality index vs. Visualized activation map	75.0%	84.0%	54.5%	0.40

### 3.4 SEEG electrical stimulation and Resective surgery verification

Among 19 patients who underwent SEEG surgery, deep electrodes passing through the left Broca’s area (*n* = 6) or left Wernicke’s area (*n* = 10) were placed in 16 patients. Among them, 4 positive electrodes were found after electrical stimulation of the left Broca’s area (3 cases were judged as bilateral Broca’s area dominance by visualization, 1 case as left dominance), and 2 cases had no language dysfunction (1 case was judged as bilateral Broca’s area dominance by visualization, 1 case as left dominance), with a positive rate of 66.7% (4/6) for Broca’s area electrical stimulation. Only 1 positive electrode was found after electrical stimulation of the left Wernicke’s area (judged as right dominance by visualization), and 9 cases had no language dysfunction (5 cases were judged as bilateral Wernicke’s area dominance by visualization, 4 cases as right dominance), with a positive rate of 10.0% (1/10) for Wernicke’s area electrical stimulation. The positive rate of electrical stimulation in the left Broca’s area was significantly higher than that in the Wernicke’s area, and the difference was statistically significant (Fisher’s exact test, *p* = 0.037).

**Figure 5:**
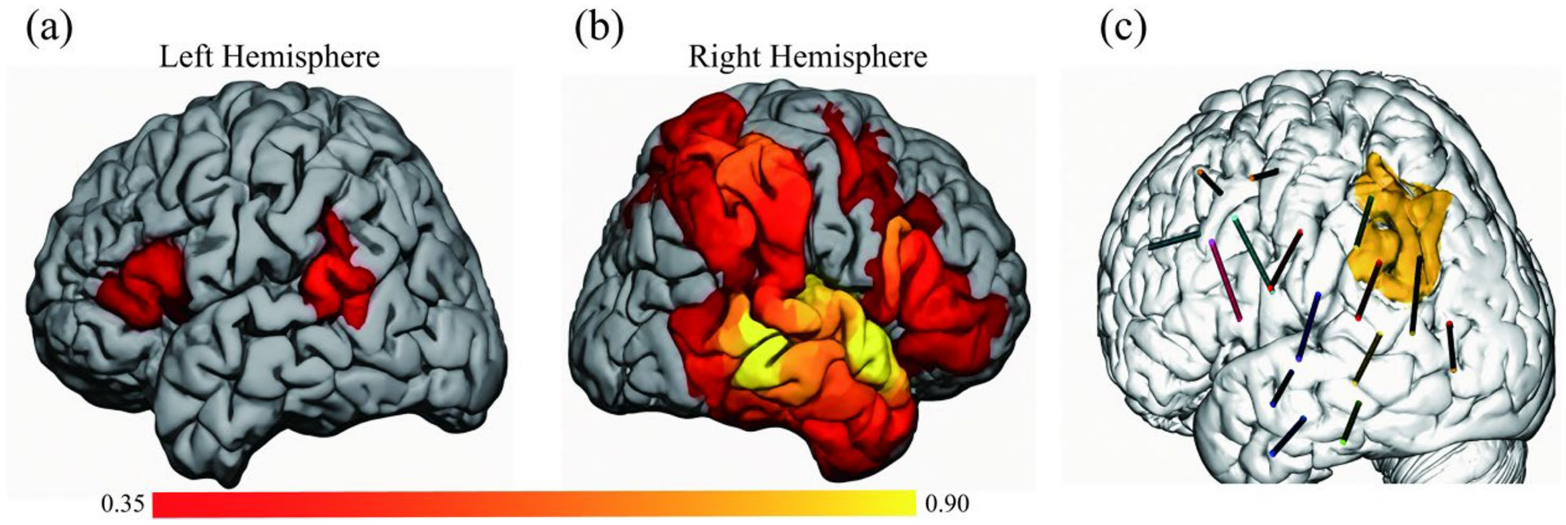
Multimodal validation of language lateralization in a representative patient. **(a)** Left hemispheric language area mapping derived from resting-state functional MRI (rs-fMRI); **(b)** Right hemispheric language area mapping derived from rs-fMRI in the same patient. The color scale ranges from 0.35 to 0.90, representing statistically significant positive functional connectivity strength, with brighter colors indicating stronger connectivity. **(c)** Surgical validation in this patient: the yellow highlighted region indicates the surgical resection range. Electrical stimulation of the left supramarginal gyrus and angular gyrus did not elicit any language deficits, and no permanent or transient postoperative language impairment was observed after resection of the indicated region.

Among the patients who received surgical treatment, 6 cases underwent resection of part of the left Wernicke’s area, of which 5 cases were judged as right Wernicke’s area dominance by visualization and 1 case as bilateral dominance. No temporary or long-term language dysfunction occurred in all patients after surgery, and postoperative pathology showed neuronal damage in the original functional areas. A representative case is shown in Figure 5.

## 4 Discussion

### 4.1 Remodeling of language lateralization in epilepsy patients

This study focused on epilepsy patients with left-sided epileptogenic foci and confirmed that the proportion of non-classical language dominance in these patients (69.4%) was significantly higher than that in healthy people (45.5%). Even in the right-handed subgroup, the proportion of non-dominant in epilepsy patients was still higher than that in healthy people, which was consistent with the research results of Pataraia et al., suggesting that the presence of epileptogenic foci can induce significant remodeling of language lateralization (29–32). Abnormal discharge of neurons in epilepsy patients can spread to the core language areas through the neural network, leading to impaired function of the left classical language areas. To compensate for language function, the brain initiates brain plasticity mechanisms, resulting in the shift of language dominant hemisphere to the contralateral homotopic area or the development of bilateral dominance, which is the core mechanism of language lateralization remodeling. This is consistent with the research results of Billot and Kiran (33).

In addition, this study found that the language dominance of Broca’s area and Wernicke’s area in epilepsy patients may be on different sides (e.g., left Broca’s area dominance accompanied by right Wernicke’s area dominance). 50% of patients with epileptogenic foci in the left frontal lobe had non-dominant in Broca’s area, while 81.8% of patients with epileptogenic foci in the left temporoparietal lobe had non-dominant in Wernicke’s area, suggesting that Wernicke’s area is more susceptible to the influence of epileptic discharge and changes in language lateralization occur. The discovery of this regional specific change is consistent with the view advocated by Hassanzadeh et al. that language areas need to be evaluated separately (23, 24). Epilepsy surgery often involves the precise resection of a single language brain area, and relying solely on the laterality index is difficult to reflect regional functional differences, which may lead to deviations in surgical decision-making. Therefore, partition evaluation has more clinical guiding value. When the resection scope of epileptogenic foci involves the left Broca’s area, Wernicke’s area and the right homotopic area, preoperative evaluation of language function is required to protect the patient’s language function.

### 4.2 Clinical application value of combined evaluation of coefficients and visualized activation maps

Visualized activation maps can intuitively guide surgery—even if the classical language areas are resected, as long as the contralateral language areas are compensatory (dominance shown by visualization), language function can still be retained after surgery, which is consistent with the view of Benke et al. who took visualization as the core evaluation index (3). The core advantage of qualitative data from visualized activation maps is that they can provide more intuitive and reliable display results of language dominant hemisphere. However, visual evaluation depends on the subjective judgment of observers, and there may be differences in results among different readers, which requires joint research and judgment by the surgical team (10). Complementary to this, the laterality index and regional laterality index provide objective and repeatable evaluation results by quantifying the functional connectivity lateralization of Broca’s area and Wernicke’s area. In this study, the consistency between the overall laterality index and visualized activation maps reached 83.3%, and the regional laterality index can accurately reflect the regional functional differences between Broca’s area and Wernicke’s area, which is more in line with the needs of clinical individualized surgery.

The combined application of the global laterality index, regional laterality index, and visualized activation map constructs a dual evaluation system of “qualitative intuition + quantitative precision” and achieves the complementary advantages of qualitative and quantitative evaluation: when the epileptogenic foci are different from the language dominant hemisphere obtained by the combined evaluation, the epileptogenic foci can be resected directly on the premise of preserving the language dominant hemisphere, avoiding invasive SEEG functional area localization and reducing the medical trauma and cost of patients; When the epileptogenic foci are on the same side as the language dominant hemisphere, but the combined evaluation shows that the adjacent language areas have had a dominant hemisphere shift (e.g., left temporal lobe epileptogenic foci accompanied by right or bilateral Wernicke’s area dominance), the resection scope of epileptogenic foci can be appropriately expanded on the premise of ensuring that the core language areas are not damaged. In the present study, partial resection of the left Wernicke’s area was performed in 6 patients with right or bilateral Wernicke’s area dominance, and no temporary or permanent language dysfunction occurred postoperatively, which strongly validates the safety of expanding the resection range. This combined evaluation scheme provides a new non-invasive method for preoperative lateralization of language areas in epilepsy, which has important clinical application value.

### 4.3 SEEG electrical stimulation and surgical verification: Core function retention in the left Broca’s area and functional weakening in the left Wernicke’s area

This study found through SEEG electrical stimulation and postoperative follow-up verification that there are significant differences in the functions of the left Broca’s area and Wernicke’s area, which provides key clinical basis for the formulation of surgical plans. For Broca’s area, even if the rs-fMRI visualized activation map is judged as bilateral dominance, electrical stimulation of the left Broca’s area mostly shows positive reactions, suggesting that bilateral dominance indicated by rs-fMRI is not equivalent to functional equalization, and the left Broca’s area still retains the core motor language function, which is consistent with the physiological function of Broca’s area as the language output center. Therefore, in epilepsy surgery, regardless of the language dominant hemisphere shown by preoperative evaluation, the left Broca’s area and its surrounding key neural network connections should be preserved as much as possible. If the epileptogenic foci are closely related to the left Broca’s area, methods such as task-based fMRI, Wada test or intraoperative awake craniotomy can be added for further evaluation to avoid irreversible motor aphasia caused by resection or injury.

For Wernicke’s area, when rs-fMRI is judged as bilateral dominance or right dominance, electrical stimulation of the left Wernicke’s area is mostly negative, and 6 patients who underwent resection of part of the left Wernicke’s area had no language dysfunction after surgery. Combined with the abnormal neuronal changes shown by postoperative pathology, it is confirmed that the function of the left Wernicke’s area has been significantly weakened. This suggests that in surgery, if the preoperative combined evaluation shows the left Wernicke’s area is non-dominant, the resection boundary can be appropriately expanded according to the scope of epileptogenic foci on the premise of ensuring that the surrounding important structures (such as arcuate fasciculus, superior temporal gyrus, etc.) are not damaged, so as to improve the total resection rate of epileptogenic foci and the clinical cure rate of surgery.

### 4.4 Influence of different data processing methods on the results of language dominant hemisphere obtained by Rs-fMRI

This study obtained through seed point-based rs-fMRI analysis that the proportion of left language dominance in right-handed healthy people was 55.5%, which was lower than the more than 80% reported by the Wada test. This difference may be due to two aspects: first, most of the healthy control subjects were medical staff with high hand flexibility and intellectual level, who may themselves have a higher proportion of atypical language dominance (34); second, rs-fMRI is more likely to capture slight differences in bilateral functional connectivity than the Wada test, and has higher sensitivity in identifying non-dominant. Rs-fMRI for language lateralization research mainly includes seed point-based analysis, independent component analysis (ICA) and graph theory analysis, and language lateralization results derived from rs-fMRI show considerable analytic variability across different analytical approaches. This study adopted the seed point-based method and found that the proportion of left language dominance in epilepsy patients was 30.6%; while Rolinski et al. used the ICA method and their studies showed that this proportion fluctuated between 26 and 75% (12, 35, 36). Among these rs-fMRI analytical strategies, the seed point-based analysis shows superior clinical utility and reliability for presurgical language lateralization evaluation. Seed point-based analysis focuses on the functional connectivity of preset core language areas, and the results are more targeted; ICA separates language network components through a data-driven approach, which is easily affected by network decomposition parameters; graph theory analysis focuses on the network topological structure and has weak direct pointing to the language dominant hemisphere. This study selected the seed point-based method, and ensured the correlation between the evaluation results and the clinical localization of language areas by defining 12 bilateral frontotemporal language-related seed points, providing a more direct reference for surgical decision-making.

## 5 Limitations

This study has several limitations. First, the sample size was relatively small, especially for specific subgroups. Larger multicenter prospective studies are needed to validate our findings, though these limitations do not undermine the core conclusions. Second, SEEG cortical stimulation mapping, the gold standard, has inherent limitations including electrode coverage constraints and potential bias from stimulation parameters and inter-observer differences. Third, only seed point-based analysis was used for rs-fMRI processing, without comparison with other methods like ICA or graph theory, which may affect language lateralization interpretation.

## 6 Conclusion

Epilepsy patients with left-sided epileptogenic foci have obvious remodeling of language lateralization, with a significantly higher proportion of non-classical language dominance than healthy people, and Wernicke’s area is more prone to dominant hemisphere shift than Broca’s area, and some patients may show crossed dominance characteristics. Bilateral language dominance indicated by rs-fMRI is not equivalent to functional equalization; the left Broca’s area still retains core language functions and needs to be prioritized for protection during surgery, while the function of the left Wernicke’s area is significantly weakened in the state of non-dominant, and the resection scope can be appropriately expanded on the premise of ensuring the surrounding important structures.

The combined evaluation system of laterality index and visualized activation maps realizes the complementary advantages of qualitative and quantitative evaluation, and has a moderate consistency with the clinical gold standard. It can provide a reliable non-invasive basis for preoperative lateralization of language areas in epilepsy, help optimize individualized surgical plans, reduce the risk of postoperative language impairment, improve the therapeutic effect of surgery, and has important clinical transformation value.

## Data Availability

The raw data supporting the conclusions of this article will be made available by the authors, without undue reservation.
